# Monocytes are essential for inhibition of synovial T-cell glucocorticoid-mediated apoptosis in rheumatoid arthritis

**DOI:** 10.1186/ar2582

**Published:** 2008-12-19

**Authors:** Dimitrios Makrygiannakis, Shankar Revu, Petra Neregård, Erik af Klint, Omri Snir, Cecilia Grundtman, Anca Irinel Catrina

**Affiliations:** 1 Department of Rheumatology, Karolinska University Hospital and Karolinska Institutet, Stockholm, S-17176, Sweden

## Abstract

**Introduction:**

Rheumatoid arthritis (RA) is characterized by synovial inflammation with local accumulation of mononuclear cells such as macrophages and lymphocytes. We previously demonstrated that intra-articular glucocorticoids decrease the synovial tissue (ST) T-cell population and therefore aimed to investigate whether this is mediated through modulation of apoptosis.

**Methods:**

Apoptosis and cell phenotype were evaluated by immunohistochemistry and dual-immunofluorescence in synovial biopsy sections from 12 RA patients before and after a mean of 11 days of an intra-articular triamcinolone knee injection. *In vitro*, RA synovial fluid (SF)-derived T cells were evaluated for Annexin V expression by multicolor flow cytometry after 24-hour exposure to dexamethasone, methylprednisolone, or triamcinolone. We also tested induction of apoptosis by dexamethasone on psoriatic arthritis SF-derived T cells using the same method.

**Results:**

Intra-articular glucocorticoids reduced ST T cells but not macrophage number. ST apoptosis levels were unchanged following treatment, virtually absent from lymphoid aggregates, and minimal in CD3^+ ^cells both before and after treatment. RA SF T cells were resistant to glucocorticoid-induced apoptosis when cultured in the presence of monocytes but were rendered sensitive to all three tested compounds upon SF isolation. Furthermore, transwell coculture of monocytes and T cells demonstrated that soluble factor(s) and not cellular contact are essential for T-cell resistance to glucocorticoid-mediated apoptosis. This feature is RA-specific as far as dexamethasone-induced apoptosis in nonisolated SF T cells obtained from psoriatic arthritis patients is concerned.

**Conclusions:**

We demonstrate that monocytes rescue synovial T cells from glucocorticoid-induced apoptosis, a feature that is specific for RA. To overcome this, we propose the use of monocyte-targeted therapies rather than T-cell apoptosis-inducing therapies.

## Introduction

Rheumatoid arthritis (RA) is a chronic inflammatory disease that is characterized by excessive synovial infiltration and proliferation of mononuclear cells (MCs) partly due to a defective apoptotic process [1]. RA synovial T cells express a phenotype suggesting chronic immune activation but have been found to be anergic [2] and resistant to apoptosis [3,4]. It has been suggested that factors such as chronic exposure to tumor necrosis factor (TNF) [5], exposure to interleukin-2 receptor (IL-2R) γ chain cytokines, and inhibitory signals received through interaction with stromal cells [3] might contribute to the T cell-specific phenotype of the rheumatoid synovium. This phenotype has been associated with the overexpression of two intracellular molecules, Bcl-2 and Bcl-xl [3,6,7], capable of blocking mitochondria-induced apoptosis.

Glucocorticoids are potent anti-inflammatory agents that modulate apoptosis of immune cells. Glucocorticoid activities can be divided in (a) genomic effects mediated through cytosolic glucocorticoid receptors (GRs) that need hours to become evident at the cellular and tissue levels and (b) nongenomic effects mediated through membrane-bound GR or nonspecific physicochemical interaction with the cell membrane which might explain some of the immediate effects observed with glucocorticoid administration *in vivo *[8]. One of the classic effects of glucocorticoids is induction of apoptosis. *In vitro*, synthetic glucocorticoids induce apoptosis of human thymocytes and activated T cells of human peripheral blood [9,10]. The mechanism of T-cell glucocorticoid-induced apoptosis is primarily mediated through the mitochondrial cell death pathway [11] and is thought to be essentially dependent on genomic effects [12]. Two of the main mechanisms for resistance to glucocorticoid apoptosis are defects in the GR signaling and/or defects of the cell apoptotic machinery, such as disregulation of the Bcl-2 rheostat [13]. To date, several synthetic glucocorticoids such as triamcinolone (for local intra-articular administration) and methylprednisolone (for both local and systemic administration) are currently used in clinical practice. Differences in the mechanisms of action of these two compounds have been previously reported [14].

We have previously demonstrated that treatment with intra-articular glucocorticoids reduces the number of synovial tissue (ST) T cells in a wide range of arthritis types and suggested that this finding might be the consequence of reduced inflammatory cell trafficking to the joints [15]. However, apoptosis induction by glucocorticoids might be an additional mechanism. In this study, we used sequential arthroscopic biopsies to characterize the effect of glucocorticoids on synovial cellularity and apoptosis levels in patients with RA. We further investigated *ex vivo *the link between synovial-derived immune cell interactions and sensitivity to glucocorticoid-induced apoptosis. We demonstrate that monocytes rescue synovial T cells from glucocorticoid-induced apoptosis through a soluble factor(s)-mediated mechanism, a feature that is specific for RA.

## Materials and methods

### Patients

Twelve patients (10 women and 2 men with a median age of 57 years and range of 34 to 83 years) with active knee arthritis (mean duration of current knee arthritis episode of 2 months and mean disease duration of 84 months) who fulfilled the 1987 American College of Rheumatology criteria for RA [16] were recruited for this study. All patients received an intra-articular injection of 40 mg of triamcinolone hexacetonide. Synovial biopsy samples from areas close to cartilage were obtained prior to and a median of 11 days (range of 8 to 14 days) after injection. All other associated treatments (including disease-modifying drugs, biologic agents, nonsteroidal anti-inflammatory drugs, and oral glucocorticoids) were maintained at constant levels for at least 2 weeks before and throughout the whole study period. The ethics committee at the Karolinska University Hospital (Stockholm, Sweden) approved all experiments on human cells and tissues. Informed consent was obtained from all study subjects.

### Tissue preparation and immunohistochemical analysis

Serial cryostat sections (7 μm) were fixed for 20 minutes with 2% (vol/vol) formaldehyde or for 10 minutes with 100% acetone and stored at -70°C. We evaluated synovial apoptosis using the TUNEL (terminal deoxynucleotidyl transferase-mediated dUTP-biotin nick end-labeling) technique and staining for the active form of caspase-3 in 2% formaldehyde-fixed sections as previously described [17]. We characterized the ST-cell phenotype in acetone-fixed sections using the following primary antibodies: mouse IgG1 anti-human CD3 (SK7; BD Bioscences, San Jose, CA, USA), mouse IgG1 anti-human CD68 (KP1; DakoCytomation, Glostrup, Denmark), and mouse IgG1 anti-human CD163 (Ber-MAC3; DakoCytomation) as previously described [17]. Matched controls were included for all markers.

### Immunofluorescence staining

Two percent formaldehyde-fixed sections were first developed with a fluorescein-labeled TUNEL kit (11684817910; Roche, Basel, Switzerland) for 1 hour at 37°C. Sections were further incubated with the polyclonal rabbit anti-human CD3 antibody (A0452; DakoCytomation) for 3 hours followed by the addition of secondary biotinylated goat anti-rabbit antibody (S0123; Vector Laboratories, Burlingame, CA, USA), which was followed by the addition of streptavidin-conjugated rhodamin red (61751; Jackson ImmunoResearch Laboratories, Inc., West Grove, PA, USA). Sections were mounted with Mowiol 4–88 mounting medium (475904; Calbiochem, now part of EMD Biosciences, Inc., San Diego, CA, USA).

### Microscopic analysis

Stained synovial biopsy sections were evaluated semiquantitatively using a four-point scale (previously described in [17]) by two independent observers (AIC and DM) who were unaware of patient identity and biopsy sequence. For quantification, synovial expression of each marker was evaluated by computer-assisted image analysis by a single observer (DM) unaware of the identity of each section (50 mean microscopic fields and a magnification of × 250), and the results were expressed as the percentage of positive stained area per total tissue area. For quantification of immunofluorescence stainings, a single observer (DM), unaware of the identity of each section, counted TUNEL/CD3 double-positive cells per total number of CD3^+ ^cells.

### Cell preparation and flow cytometric analysis

Synovial fluid (SF) MCs from 11 RA and 2 psoriatic arthritis patients were isolated by gradient centrifugation using Ficoll-Paque (Pharmacia, Uppsala, Sweden) and stored in liquid nitrogen until assayed. SF MCs were cultured in triplicate in RPMI supplemented with 2 mM glutamine, 100 IU/mL penicillin and streptomycin, and 20% heat-inactivated fetal calf serum (all from Gibco, now part of Invitrogen Corporation, Carlsbad, CA, USA) and incubated at 37°C in a humidified atmosphere containing 5% CO_2_. Dexamethasone (861871; Sigma-Aldrich, St. Louis, MO, USA) was added to the cultures at final concentrations of 10, 1,000, or 10,000 nM and incubated for 24 hours. In four similarly processed RA SF MC samples, triamcinolone hexacetonide (Lederspan; Meda AB, Stockholm, Sweden) and methylprednisolone acetate (Depo-Medrol; Pfizer Inc, New York, NY, USA) were added at final concentrations of 50, 5,000, or 50,000 nM and incubated for 24 hours. To test whether glucocorticoids are able to induce apoptosis of SF-derived T cells, SF MCs processed as described were stained with mouse IgG2b peridin chlorophyll protein-conjugated anti-CD14 antibody (340585; BD Biosciences) and with mouse IgG1 phycoerythrin-conjugated anti-CD3 antibody (HIT3a; BD Biosciences), followed by incubation with Annexin V (TA5532; R&D Systems, Minneapolis, MN, USA) and flow cytometry analysis. T cells were identified based on scatter properties and CD3 expression and were analyzed for expression of Annexin V.

### Synovial fluid T-cell isolation and flow cytometric analysis

To test the effect of glucocorticoids on isolated T cells derived from the SF, we used a negative selection isolation method (Pan T Cell Isolation Kit II human; Miltenyi Biotec, Bergisch Gladbach, Germany) that resulted in a cell purity of more than 90% as tested by flow cytometry with a phycoerythrin-conjugated IgG1 mouse anti-human CD3 antibody (HIT3a; BD Biosciences). Isolated RA T cells were cultured in triplicate in the same medium as SF MCs and incubated for 24 hours with previously mentioned doses of dexamethasone (n = 7), triamcinolone (n = 4), or methylprednisolone (n = 4). Cells were then stained with mouse IgG1 allophycocyanine-conjugated anti-CD3 antibody (555335; BD Biosciences) and incubated with Annexin V and 7-amino-actinomycin D (7-AAD) as specified by the manufacturer (559763; BD Biosciences) and analyzed by flow cytometry. T cells were gated as CD3^+ ^cells, and apoptosis was quantified as the mean percentage of Annexin V^+ ^cells from the total number of gated cells.

### Transwell coculture experiments

SFs from four additional RA patients were used for transwell coculture experiments. T cells and monocytes were isolated through positive selection using human CD3 and CD14 microbeads (Miltenyi Biotec) in accordance with manufacturer instructions, resulting in a cell purity of more than 92% as tested by flow cytometry with mouse IgG1 fluorescein isothiocyanate (FITC)-conjugated anti-human CD3 (555332; BD Biosciences) and mouse IgG2b FITC-conjugated anti-human CD14 antibody (345784; BD Biosciences). Isolated CD3 and CD14^+ ^cells were cocultured in duplicates on transwell permeable culture plates (pore size of 0.4 μM) (3450; Corning Life Sciences, Acton, MA, USA) in the same medium as SF MCs and incubated for 24 hours with or without dexamethasone (1,000 nM/mL). T cells from coculture were then stained with mouse IgG1 allophycocyanine-conjugated anti-human CD3 antibody (555335; BD Biosciences), followed by incubation with Annexin V and 7-AAD as specified by the manufacturer (559763; BD Biosciences) and analyzed by flow cytometry. T cells were gated as CD3^+ ^cells, and apoptosis was quantified as the mean percentage of Annexin V and 7-AAD^+ ^cells from the total number of gated cells.

### Statistical analysis

Statistical analysis was performed using the Wilcoxon test followed by Bonferroni correction for multiple comparisons of paired samples for the synovial biopsy data. *In vitro *data were analyzed by one-way analysis of variance followed by Tukey *post hoc *analysis or nonparametric Wilcoxon for paired samples when appropriate. *P *values of less than 0.05 were considered statistically significant.

## Results

Clinical response following intra-articular glucocorticoids is accompanied by a decrease in the number of ST T cells. All patients included in the study were clinical responders as evaluated by physician assessment during arthroscopies. The clinical response was paralleled by a significant decrease in the number of ST T cells (from a mean ± standard error of the mean [SEM] of 15.9 ± 4.1 to a mean ± SEM of 5.4 ± 1.9), as evaluated by CD3 staining without changes in the number of ST macrophages, as evaluated by both CD68 and CD163 staining (data not shown).

**Figure 1:**
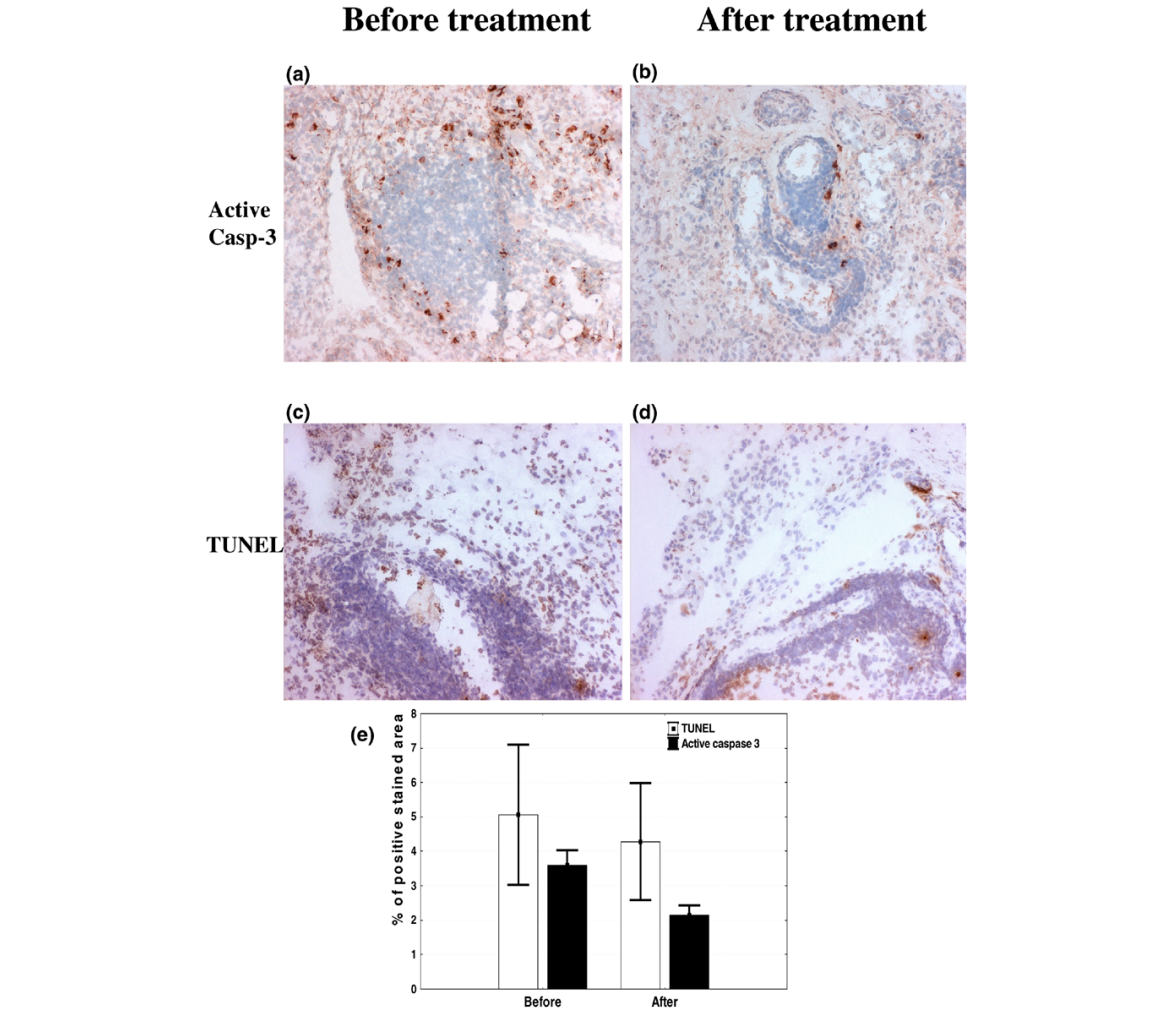
**Intra-articular glucocorticoids do not increase synovial tissue apoptosis levels in rheumatoid arthritis**. Frozen sections of rheumatoid arthritis synovial biopsy tissues (n = 12) show aminoethylcarbazole staining (red) for active caspase-3 (hematoxylin-counterstained) before **(a) **and after **(b) **treatment and diaminobenzidine staining (brown) for TUNEL (hematoxylin-counterstained) before **(c) **and after **(d) **treatment (original magnification × 125). **(e) **Results from image analysis of synovial biopsy sections for active caspase-3 and TUNEL staining before and after intra-articular corticosteroid injection. Values represent the mean ± standard error of the mean. TUNEL, terminal deoxynucleotidyl transferase-mediated dUTP-biotin nick end-labeling.

**Figure 2:**
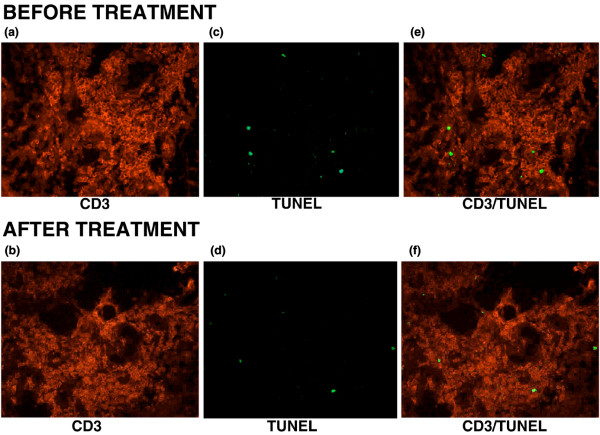
**CD3^+ ^synovial T cells exhibit minimal levels of apoptosis in rheumatoid arthritis synovium both before and after intra-articular glucocorticoids**. Photomicrographs illustrate fluorescent staining of CD3^+ ^cells (red, rhodamin red) before **(a) **and after therapy **(b)**, TUNEL^+ ^cells (green, fluorescein) before **(c) **and after therapy **(d) (b, e)**, and superimposed stainings before **(e) **and after therapy **(f)** (original magnification × 320). TUNEL, terminal deoxynucleotidyl transferase-mediated dUTP-biotin nick end-labeling.

The decrease in the ST T-cell population is not mediated through apoptosis induction. Synovial apoptosis evaluated by TUNEL and staining for active caspase-3 did not show changes following intra-articular glucocorticoid injection. ST lymphoid aggregates showed absent to minimal apoptosis levels with both methods both before and after intra-articular glucocorticoid injection (Figure 1). This was confirmed by dual-immunofluorescence demonstrating minimal (<2%) levels of apoptosis (TUNEL) in CD3^+ ^cells both before and after treatment (Figure 2).

**Figure 3:**
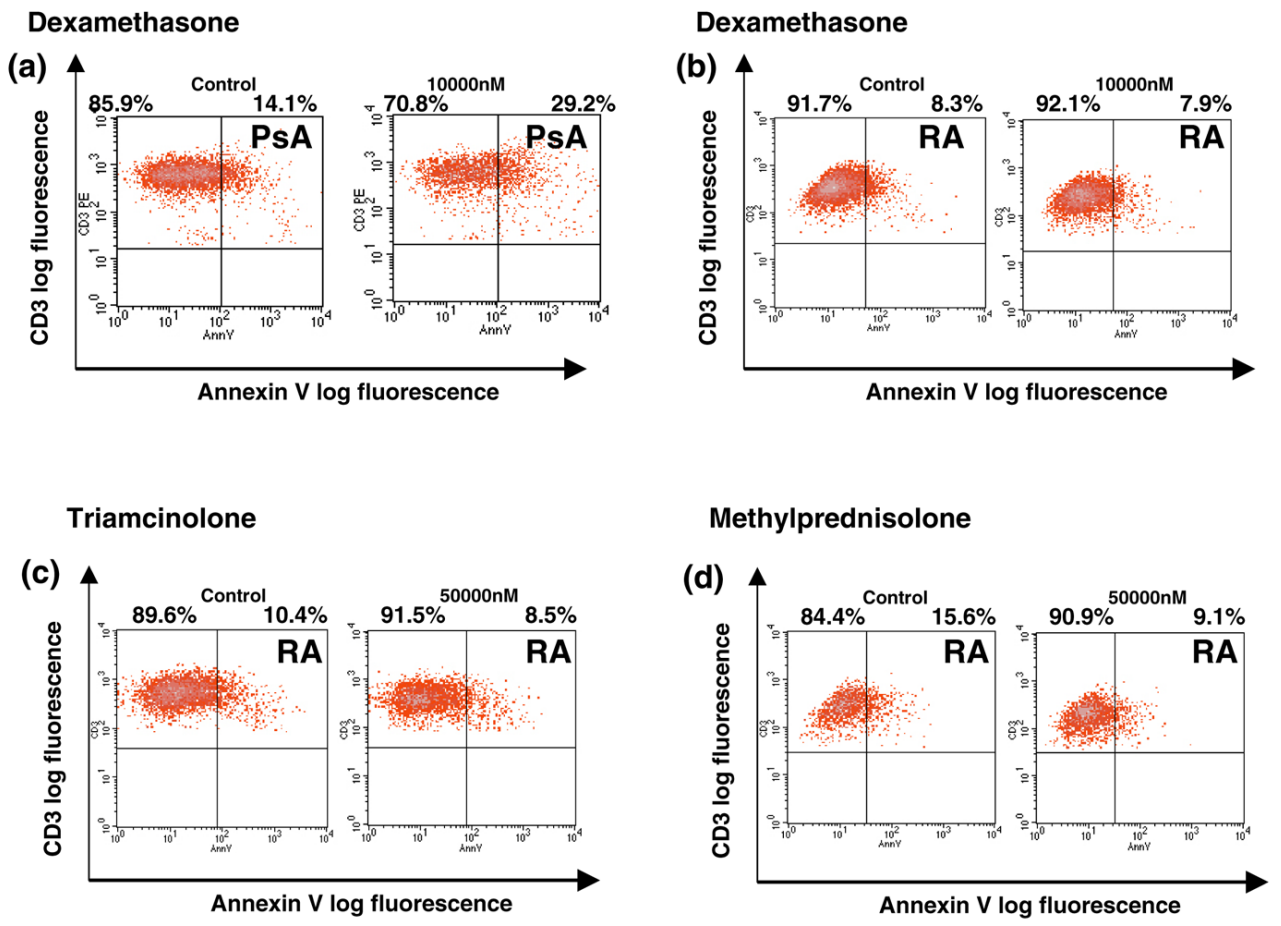
**Rheumatoid arthritis (RA)-derived, but not psoriatic arthritis (PsA)-derived, synovial fluid (SF) T cells cocultured with monocytes are resistant to glucocorticoid-induced apoptosis**. Flow cytometric analysis shows that dexamethasone induces an increase in the number of SF CD3/Annexin V double-positive T cells of PsA patients (n = 2) **(a)**, whereas dexamethasone (n = 11) **(b)**, triamcinolone (n = 4) **(c)**, and metylprednisolone (n = 4) **(d) **fail to induce similar changes in apoptosis in SF CD3^+ ^T cells of RA patients.

RA SF-derived T cells are resistant to glucocorticoid-induced apoptosis in the presence of SF-derived monocytes. To further investigate the effect of glucocorticoids on T-cell apoptosis, SF MCs containing both monocytes and lymphocytes but no fibroblast cells were incubated *ex vivo *with dexamethasone. T cells in cocultures with monocytes of RA-derived (Figure 3b) but not psoriatic arthritis-derived (Figure 3a) SF were resistant to dexamethasone-induced apoptosis. As different synthetic glucocorticoid compounds might have distinct effects, triamcinolone (Figure 3c) and methylprednisolone (Figure 3d) were also tested but both failed to induce T-cell apoptosis in monocyte-T cell cocultures derived from RA SF.

**Figure 4:**
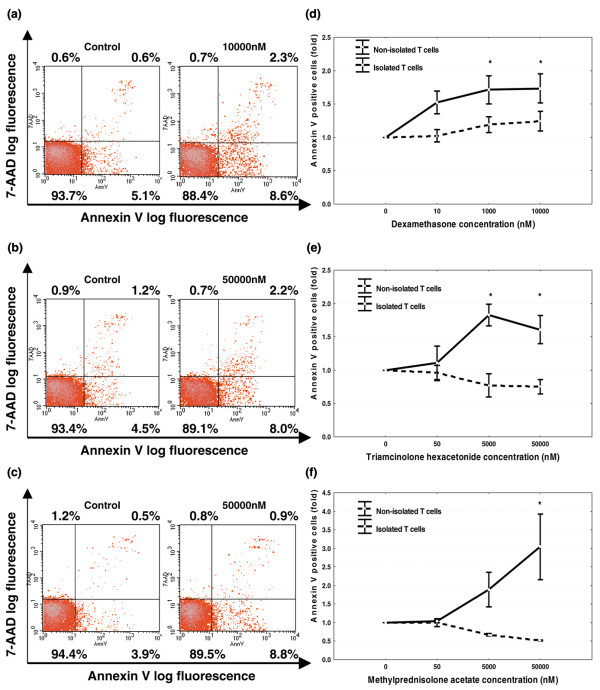
**Rheumatoid arthritis (RA) synovial fluid (SF) T cells become susceptible to glucocorticoid-induced apoptosis upon separation from monocytes**. Twenty-four-hour exposure to dexamethasone **(a)**, triamcinolone **(b)**, and metylprednisolone **(c) **of negatively isolated T cells from SF of RA patients (n = 4) increases apoptosis evaluated as Annexin V^+ ^7-AAD^- ^cells. Graphs demonstrate that all glucocorticoid compounds **(d, e, f) **induce apoptosis in isolated T cells but not in nonisolated T cells (dashed line represents nonisolated T cells and continuous line represents isolated T cells). Values are the mean ± standard error of the mean and are expressed as the ratio of Annexin V^+ ^cells in the experimental cultures to those in the control cultures (fold). *P < 0.05. 7-AAD = 7-amino-actinomycin D; AnnV, Annexin V.

T cell-monocyte interaction is essential to render RA SF T cells resistant to glucocorticoid-induced apoptosis. We hypothesized that the synovial RA environment with close contact between different subsets of inflammatory cells and presence of mediators contributes to the glucocorticoid-induced apoptosis-resistant phenotype of SF-derived T cells. To confirm this, SF-isolated T cells were treated *in vitro *with different synthetic glucocorticoid compounds. All three tested compounds at equivalent doses resulted in a significant fold increase of the apoptosis levels of isolated T cells to a maximum of 1.7 ± 0.2 for dexamethasone, 1.8 ± 0.2 for triamcinolone, and 3.0 ± 0.8 for methylprednisolone (all values expressed as mean ± SEM) (Figure 4).

**Figure 5:**
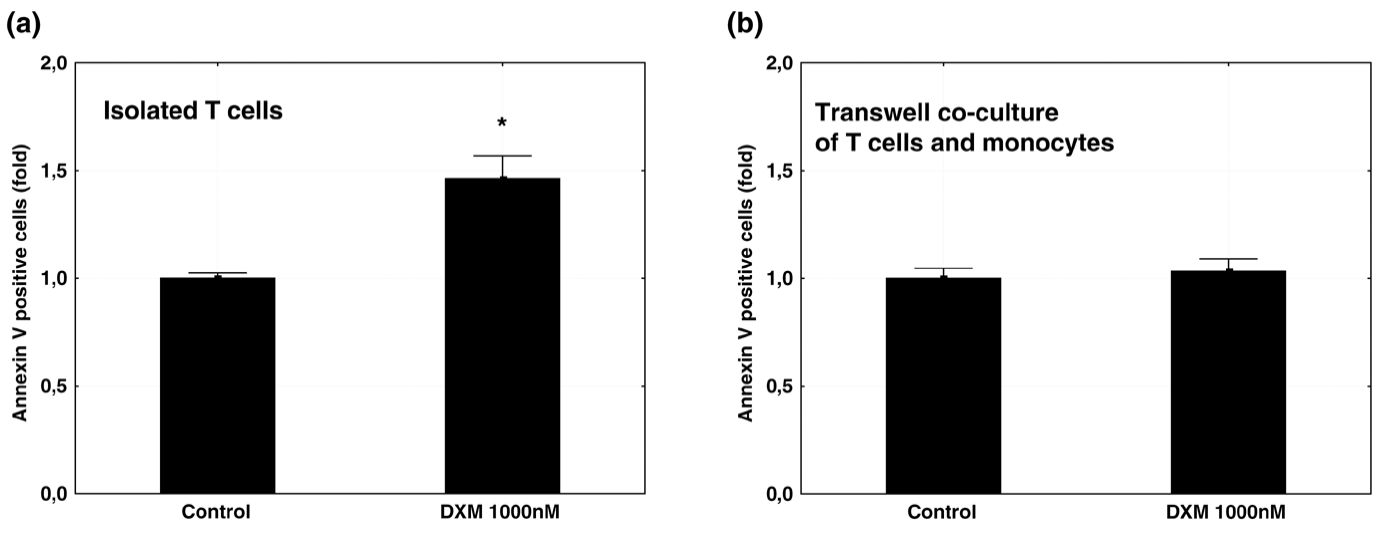
**Coculturing of T cells in the presence of, but without direct contact with, monocytes rescues isolated T cells from glucocorticoid-induced apoptosis**. Graphs demonstrate that dexamethasone induces apoptosis in isolated T cells **(a)**, an effect that disappears in the presence of monocytes **(b)**. Values are the mean ± standard error of the mean and are expressed as the ratio of Annexin V^+ ^cells in the experimental cultures to those in the control cultures (fold). *P < 0.05.

Soluble factor(s) rather than cellular interaction are essential for the induction of the T-cell apoptosis-resistant phenotype. To further investigate the mechanism responsible for the resistance of T cells to glucocorticoid-induced apoptosis, we analyzed the importance of cellular contact versus soluble factor(s). Isolated T cells were cultured in the presence of, but without direct contact with, isolated SF-derived monocytes. Incubation with dexamethasone did not result in apoptosis of the T cells (mean ± SEM of 1.0 ± 0.1-fold increase as compared with control), suggesting that soluble factor(s) rather than cellular contact are primarily responsible for induction of the apoptosis-resistant phenotype of the RA synovial T cells (Figure 5).

## Discussion

Intra-articular glucocorticoids are a powerful adjuvant therapy for a variety of inflammatory joint diseases which efficiently reduces local joint inflammation. We demonstrate here that, in RA patients, this effect is mediated through the reduction of the synovial T-cell population as previously suggested in a cohort of patients with arthritis of different pathogenesis [18]. Furthermore, we provide evidence for the first time that RA-derived synovial T cells are resistant to apoptosis induction by glucocorticoids due to a soluble factor(s)-mediated interaction with monocytes.

Our immunohistochemistry results demonstrate that local administration of glucocorticoids decreases the number of lymphocytes without changes in the monocyte/macrophage population, evaluated as both CD68^+ ^and CD163^+ ^cells. The T cell-specific effect of locally administrated glucocorticoids might reside in the imbalance between the two alternatively spliced transcripts of the GR that have been suggested to have different functional characteristics. Exposure of cells to proinflammatory stimuli such as TNF and IL-1 can lead to induction of β-isoform of human glucocorticoid receptor (hGRβ) and suppression of hGRα, resulting in diminished glucocorticoid responsiveness [19]. Furthermore, within the same tissues, the levels of hGRβ may vary considerably between different types of cells [20]. Thus, the local proinflammatory milieu in an inflamed joint might contribute to the cell type-specific effect of locally administrated glucocorticoids.

Our findings suggest a distinct effect of local as compared with systemic administration of glucocorticoids which has been shown to decrease both lymphocyte and macrophage populations [21]. The difference might reside in the use of distinct synthetic glucocorticoid compounds for local versus systemic administration (that is, triamcinolone versus prednisolone/methylprednisolone). It has been suggested that, at equivalent doses, the effects of triamcinolone and dexamethasone, but not of methylprednisolone, are suppressed by overexpression of the hGRβ that acts as a natural dominant negative inhibitor of the transactivation of glucocorticoid-responsive genes [14]. However, when we tested the three compounds at equivalent doses, we did not observe differences in the *in vitro *effect of any of the compounds in any cell population studied. An alternative explanation is the apparently specific effect of systemically as compared with locally administrated high-dose glucocorticoids to induce profound monocytopenia in the peripheral blood [22] that would interfere with local synovial accumulation of monocytes/macrophages.

The observed reduction in the number of synovial T cells might be due either to a lower rate of recruitment or to a higher rate of clearance at the site of inflammation. We have previously demonstrated that intra-articular glucocorticoids decrease synovial expression of ICAM-1 (intracellular adhesion molecule-1), an adhesion molecule essential for leukocyte migration, despite minimal changes in the inflammatory phenotype of the endothelial synovial cells [15]. Our current results showing resistance of RA synovial T cells to glucocorticoid-induced apoptosis provide further indirect support for decreased leukocyte recruitment as the major mechanism responsible for the decreased cellularity observed after treatment with intra-articular glucocorticoids.

In RA, synovial-derived T cells have a phenotype suggestive of chronic immune activation but express low levels of cytokines and show signs of anergy [2]. These cells are resistant to apoptosis, partly due to their interaction with other cell populations present in the RA synovial inflamed milieu. It has been previously demonstrated that synovial-derived isolated T cells are rescued from spontaneous apoptosis through an integrin-ligand interaction with stromal cells, an effect that was mimicked by the addition of several members of the IL-2R γ chain cytokines, such as IL-15 [3]. Along the same line, coculture of autologous synovial RA T cells with monocytes induces homeostatic proliferation of T cells which is dependent on the membrane-bound TNF on monocytes [23]. We demonstrate that not only spontaneous but also glucocorticoid-induced apoptosis is dependent on the complex cell-cell interaction in the rheumatoid synovium. The essential factor in this situation appears to be the T cell-monocyte interaction to the extent that T-cell isolation renders the cells sensitive to apoptosis, while coculture of T cells with monocytes in the absence of fibroblasts prevented the effect of all tested glucocorticoid compounds. Furthermore, we propose that the main mechanism by which monocytes are able to rescue T cells is a soluble factor(s)-mediated interaction rather than cell-cell contact. It has been demonstrated, for example, that monocytes isolated from RA SF express IL-15 [24], a cytokine able to upregulate Bcl-2 expression [3] and to render activated T cells resistant to glucocorticoid-mediated apoptosis [25]. The mechanism appears to be RA-specific given that T-cell apoptosis induction was observed in cocultures of cells obtained from psoriatic arthritis in the presence of dexamethasone at similar doses.

## Conclusion

We demonstrate that monocytes are essential in rescuing synovial T cells from glucocorticoid-induced apoptosis through a soluble factor(s)-mediated mechanism, a feature that is specific for RA-derived synovial T cells. We propose that this might be overcome by the combination of locally administrated glucocorticoids with monocyte-targeted therapies rather than T-cell apoptosis-inducing therapies.

## Abbreviations

7-AAD: 7-amino-actinomycin D; FITC: fluorescein isothiocyanate; GR: glucocorticoid receptor; hGR: human glucocorticoid receptor; IL: interleukin; IL-2R: interleukin-2 receptor; MC: mononuclear cell; RA: rheumatoid arthritis; SEM: standard error of the mean; SF: synovial fluid; ST: synovial tissue; TNF: tumor necrosis factor; TUNEL: terminal deoxynucleotidyl transferase-mediated dUTP-biotin nick end-labeling.

## Competing interests

The authors declare that they have no competing interests.

## Authors' contributions

DM performed the immunohistochemistry and flow cytometry experiments, participated in acquisition, analysis, and interpretation of data, and drafted the manuscript. SR designed, performed, and analyzed the transwell experiments and participated in interpretation of the data and writing of the manuscript. PN and EK recruited the patients for the study, performed arthroscopies, and participated in acquisition and interpretation of the data. OS participated in the flow cytometry experiments. CG participated in acquisition and analysis of the data and drafting of the manuscript. AIC conceived the study, participated in its design and coordination, analyzed the data, and helped to draft the manuscript. All authors read and approved the final manuscript.
